# Transferring the critically ill patient: are we there yet?

**DOI:** 10.1186/s13054-015-0749-4

**Published:** 2015-02-20

**Authors:** Joep M Droogh, Marije Smit, Anthony R Absalom, Jack JM Ligtenberg, Jan G Zijlstra

**Affiliations:** Department of Critical Care, Research Program for Critical Care, Anesthesiology, Per-operative and Emergency medicine (CAPE), University Medical Center Groningen, University of Groningen, Hanzeplein 1, 9700 RB Groningen, The Netherlands; Department of Anesthesiology, Research Program for Critical Care, Anesthesiology, Per-operative and Emergency medicine (CAPE), University Medical Center Groningen, University of Groningen, Hanzeplein 1, 9700 RB Groningen, The Netherlands; Emergency Department, Research Program for Critical Care, Anesthesiology, Per-operative and Emergency medicine (CAPE), University Medical Center Groningen, University of Groningen, Hanzeplein 1, 9700 RB Groningen, The Netherlands

## Abstract

During the past few decades the numbers of ICUs and beds has increased significantly, but so too has the demand for intensive care. Currently large, and increasing, numbers of critically ill patients require transfer between critical care units. Inter-unit transfer poses significant risks to critically ill patients, particularly those requiring multiple organ support. While the safety and quality of inter-unit and hospital transfers appear to have improved over the years, the effectiveness of specific measures to improve safety have not been confirmed by randomized controlled trials. It is generally accepted that critically ill patients should be transferred by specialized retrieval teams, but the composition, training and assessment of these teams is still a matter of debate. Since it is likely that the numbers and complexity of these transfers will increase in the near future, further studies are warranted.

## Introduction

Since the establishment of the first ICUs in the 1950s, the demand for intensive care has grown exponentially. When demand exceeds supply, or when highly specialized care is required, transfer of critically ill patients becomes necessary. In the United Kingdom alone, more than 10,000 patients required secondary transfers in 1986 [1]. In the USA 1 in 20 patients requiring ICU care is transferred to another hospital [2]. Similar transfer rates probably occur elsewhere.

The number of transfers is likely to increase because of supply-demand imbalances. Recognition that centralization of specialist care is associated with reduced mortality rates might generate a new stream of transfers [2]. A recent study conducted in the USA suggested that the lives of 4,000 patients might have been saved in a year had they been transferred to another, better qualified hospital [3].

Interhospital transfers may save lives but they are expensive, logistically challenging, and risky. The transport process itself is associated with a risk of physiological deterioration and adverse events. The incidence of adverse events is proportional to the duration of the transfer, to the pre-transfer severity of illness or injury and to the inexperience of the medical escorts [4-6].

Since the late 1970s, safety concerns have motivated several studies of when, how and where to transfer critically ill patients. One of the first concluded that earlier transfer, resuscitation before transfer, continuing medical care during the journey, and hence a slower and smoother journey are beneficial to patients [7] and these conclusions apply to this day. In 1986 Ehrenwerth and colleagues [8] concluded that, with a specialized transport team and appropriate haemodynamic stabilization and monitoring, severely ill patients can be transported safely. From then on, the equipment improved, trolleys were modified and the first mobile ICU appeared [9].

Although transport guidelines appeared during the 1990s [10,11], a review published in 1999 still reported adverse events in up to 70% of transports. This led the authors to urge intensivists to follow guidelines concerning logistical organization, personnel, equipment and monitoring during transport [12]. Newer guidelines continued to emphasize the principles concerning personnel, organization and equipment [13-15]. Nonetheless, high rates of incidents continued to be published, many of which appeared to be avoidable, and associated with non-adherence to the guidelines [16-19].

In 2005, Haji-Michael [20] discussed two main reasons why, despite the existence of guidelines, interhospital transfer of the critically ill patient is still associated with avoidable mishaps. The first reason concerns sponsorship: those with responsibility and authority for the care of the patients are simply not the ones doing the transfers. The second reason is a lack of a motivation for change - we have always somehow managed [20]. A third reason might be the lack of evidence that the recommendations are of benefit. The guidelines present clear recommendations but are based on weak evidence; cohort studies, case series, and expert opinion.

In this review we evaluate the current literature on the organization and safety of transfers of critically ill adult patients. We will also draw on the literature concerning paediatric intensive care transfers, since these have already been well organized for a long time.

## Research and quality management

Transport of critically ill patients repeatedly illustrates Murphy’s Law (‘if anything can go wrong, it will’). Quality control studies and research into measures to improve safety depend strongly on accurate and reliable data. However, the reported incidence of adverse events varies from 3 to 75% [6,21], not only because of differences in incidences, but also because different definitions are used. For example, Philpot and colleagues [21] defined as incidents unintended extubation, difficult intubation, intravenous access loss, medication errors, pneumothorax and bag-valve ventilation required on arrival. Other studies also consider blown equipment fuses or transport delays to be incidents [22,23]. These different definitions make it difficult to compare incident rates.

Another problem is that it is sometimes difficult to attribute adverse events to the transport process itself due to poorly documented pre-transport variables and post-transport management differences. Furthermore, many studies only examined short-term adverse events, although it cannot be ruled out that transport-related adverse events can occur later on [24]. Incident reports with standardized definitions are of major importance for quality management as well as for research purposes.

Risk scores are used to quantify severity of illness, estimate mortality risks, and for benchmarking of ICUs. These scores are validated for a certain period after ICU admission and for a certain patient population but not for this specific selection of transferred patients [25,26]. The transfer process itself may even influence the severity score. In the paediatric literature there is evidence of changing severity scores due to stabilization and transfer by specialized retrieval teams [27,28]. Although some scores, such as Acute Physiology and Chronic Health Evaluation III and Intensive Care National Audit and Research Centre score, adjust for admission source [29,30], lead time bias as well as other undefined influences are still thought to explain differences in severity scores after transfer [25,31]. Therefore, it is not possible to make realistic outcome comparisons between transferred and non-transferred patients by using our standard scoring systems [32,33]. Research based on scoring systems that are not validated for this specific patient population will not lead to reliable conclusions.

Severity scores as a guide for triage for the necessary level of expertise of a transfer team has been investigated in two small studies [34,35]. Although this score did seem to be very useful in discriminating between high-risk and low-risk patients, the relevance to the critically ill is doubtful since only a few of the low-risk patients were actually admitted to an ICU. Moreover, since specialized retrieval teams seem to transfer sicker patients with fewer incidents [36], scoring systems may only be of value in predicting the risk of non-expert transfers [37].

Reported studies have typically a case-series or before-and-after design while randomized controlled blinded trials are very scarce. With the inherent limitations of definitions and severity scoring systems and the complex organization, high-level evidence will still remain scarce for a long period. We have to realize this when evaluating the present literature and guidelines.

## Incident prevention

By definition, critically ill patients are prone to changes in their condition even without being transported. The goal during every transport should be the continuation of high-quality ICU care, while preventing deterioration or incidents. Incidents may be divided into medical and technical incidents (Table 1). Medical adverse events are most often cardiovascular or respiratory events. The most common cardiovascular events are hyper- and hypotension, brady- and tachycardias, and arrhythmias, with a reported incidence varying from 6% to 24%. Respiratory events are most often inadequate ventilation or oxygen desaturation with reported incidences ranging from 0 to 15% [5,19,23,36].

**Table 1:** **Incidents**

	**Medical**		
	**Cardiovascular**	**Respiratory**	**Technical**
Incidence	6-24%	0-15%	9-36%
Common events	Hypo-/hypertension	Inadequate ventilation	Power failure
	Brady-/tachycardias	Oxygen desaturation	Gas supply problems
	Arrhythmias		Missing equipment
			Damaged equipment

Up to 31% of incidents are classified as significant; up to 79% require an intervention; 52 to 91% are preventable.

Equipment failure or technical problems are common and may account for 46% of all incidents [38-40]. Reported incidences vary from 9% to 36% [23,36,41]. Transfer by specialized retrieval teams seems to lower the incidence of technical failure [41], emphasizing the need for training and technical understanding of the equipment used [22] and the need for standardized transfer equipment [20]. Of all incidents, up to 31% are classified as significant [4,38] and up to 79% require an intervention [40].

Strikingly, most incidents seem to be preventable. One study reported that up to 91% of incidents were preventable [39]. Factors associated with fewer incidents are good crew skills/teamwork, checking equipment and the patient, patient monitors and good interpersonal communication [39].

## Specialized retrieval teams versus standard transportation

In 1987 Pearl and colleagues [42] argued that the critical ill transport team is incomplete without a transport physician, just as an ICU would be incomplete without an intensivist. Since it is desirable to maintain an equivalent level of intensive care during transfer to that before transfer, it seems reasonable that a physician should accompany the patient. However, there are no published prospective randomized studies comparing a physician-staffed transport team with a non-physician-staffed team. The available evidence is of a lower level and mainly from paediatric care. Comparing 130 paediatric transports, 8% of all problems occurred with a specialized physician-staffed transport team, 20% occurred with a non-physician-staffed specialized team and 72% occurred with escorts without transport training, even though there were far more specialized physician transfers (54) than non-physician (44) and untrained escorts (32) [43]. Another study comparing transfer by air and ground transportation found significantly better protocol adherence when patients were transferred by air, which according to the authors was the result of the advanced trauma training of the attending flight physician [44]. Vos and colleagues compared 137 transports performed by referral specialists (mainly paediatricians) with 112 transports performed by a specialized retrieval team (mainly paediatric intensivists). Transfers performed by the referral physicians were associated with a higher incidence of complications, unavailability of equipment, and more frequent requirement for acute interventions upon arrival [45]. This was concordant with an earlier study by Bellingan and colleagues [46] that showed a reduction in acute physiology disturbances and a reduced mortality in critically ill patients transferred by a specialized retrieval team.

Nonetheless, a review published in 2006 concluded that insufficient data existed to determine whether the use of specialist transport personnel improves patient outcome. Of 39 publications, 33 were excluded because there was either no control group or an unsuitable control group. In only one study, intervention and control groups were matched [47]. No study was prospective, randomized and controlled. In recent years two before and after studies, performed in the same region, showed that the establishment of a specialized retrieval team was associated with a decline in adverse events (from 34 to 12.5%) [19,36]. In the first phase critically ill patients were transported by standard ambulances with or without referral specialist accompaniment, whereas in the second phase a specialized retrieval team comprising an intensivist and an ICU nurse performed the transfers. This specialized team appeared to be able to transfer sicker patients with fewer and less severe adverse events. In 2011 Kue and colleagues [48] presented similar findings in a preliminary report showing that the introduction of a specialized transport team for intrahospital transfers reduced the incidence of adverse events from 8 to 1.7%. In a large observational study of children admitted to 29 paediatric ICUs, Ramnarayan and colleagues [49] found that transfer by a specialized transfer team was associated with an odds ratio of mortality of 0.58 (95% confidence interval 0.39 to 0.87).

The obvious advantage of a specialized retrieval team is that it is more familiar with transport-specific procedures and equipment, although several other advantages of retrieval teams have also been proposed. Britto and colleagues [28] concluded that retrieval teams are better able to stabilize the patient prior to the transfer and Iwashyna [50] also argued that front-end discontinuity would be better addressed by an expert transport team.

A retrieval team may also better deal with logistic problems. It may be very difficult to maintain a sufficient amount of trained personnel in all hospitals, especially in the smaller ones. Establishing centrally located retrieval teams might then be a better option [51,52]. Moreover, these retrieval teams can also be deployed in remote and rural area health facilities to provide critical care skills to resuscitate and stabilize patients, before transferring them [53,54].

Although unequivocal evidence is not (yet) available, expert opinion is clear: critically ill patients should preferably be transferred by a specialized retrieval team. In a survey among the medical heads of all ICUs in the Netherlands published in 2008, escorting personnel and transport facilities were rated as the most important factors in considering whether or not a transfer would be feasible [55]. It is no surprise, therefore, that most intensive care societies recommend the use of specialized retrieval teams [56-58] or at least the use of specific trained personnel [14,15].

## Transport mode

Road ambulances, fixed wing aircraft and helicopters are all used for interhospital transfers. Many studies, particularly those involving secondary transfer of patients who have suffered traumatic injuries, have found that air transport was time saving [59-62]. These savings can, however, easily be offset by mobilization time (fixed wing or rotary wing aircraft are not always immediately available) and by requirement for additional ground transport between landing site and hospital.

In these studies, ground transports in the control groups were performed by local ambulance services. If ground transfers are performed by centrally located specialized transfer teams, overall transfer times may increase, since these teams must first travel from their base to the referring hospital. For this reason, Safford and colleagues [62] compared transfer times between helicopter and ground transports with specialized ambulance teams, stationed at four different bases. The time benefit for air transport was only 27 minutes.

There are no prospective randomized controlled trials showing that the (modest) reduction in transport time with air transport influences patient outcome. In 2011, a retrospective study in the United States compared interhospital transfer by helicopter and road ambulance of almost 75,000 trauma patients [60]. Helicopter transfer was only a predictor of survival for the severely injured - those with an injury severity score of >15 (odds ratio 1.09). However, helicopter crews are well trained medical teams, whereas ambulance crews sometimes lack essential critical care experience. This could explain the survival benefit and is a potential source of bias in this study. Borst and colleagues [61] recently compared outcome among almost 4,000 patients transferred by helicopter or by specialized acute life support road ambulance. With equivalent crew experience mortality rates were comparable.

It appears then that transfer mode does not affect outcome, or that units are not transferring the appropriate patients by air. Walcott and colleagues [63] found a relatively long interval between arrival and intervention in patients transferred for neurosurgical evaluation. They concluded that triage to helicopter transport was inappropriate. This study emphasizes the importance of triage for air transport prioritizing the patient that will most likely benefit from a reduced transport time. Other factors which should be taken into account are the additional costs of air transport [62], the potential risk, especially for rotary wing aircrafts, the confined space despite the need for ongoing intensive care, the adverse effects of noise and vibration on patient physiology, equipment and communication, long mobilization times and influence of weather conditions [15]. Conversely, road transport has the advantage of lower overall costs, rapid mobilization time, fewer limitations by weather conditions, less potential for physiological disturbance and easier patient monitoring and handling [15]. It has been advised to consider helicopters for transfer distances above 80 km (50 miles) and fixed wing aircraft for distances above 240 km (150 miles) but the choice for the individual patient should be based on clinical judgement [15,64,65].

## Preparation

The key to successful transport of the critically ill patient is stabilization before transport [42]. Since up to 91% of incidents are preventable [39], often by better preparation, it is no surprise that the importance of assessing, resuscitating and stabilizing a patient before transport is still emphasized [14,66].

Of course, these interventions take time to perform, but time spent undertaking intensive care interventions at the referring hospital does not worsen patient outcome [67]. These interventions have even been associated with a shorter length of hospital stay [68].

## Equipment

Over the years, multiple recommendations for minimum transfer equipment requirements have been made [11,14,15,42,69]. These focus not only on the continuation of normal critical care (like monitoring, ventilation, administering medication), but also on transfer-specific items (gas supply, batteries) and incident management (defibrillator, chest tubes). In general, an ICU monitor able to display electrocardiography, several pressure curves, capnography and oxygen saturation, a ventilator (preferably an ICU ventilator), airway management tools, arterial and central venous lines, and various medications are advised.

Equipment should be properly mounted in accordance with government regulations. Transfer trolleys should carry all the equipment, such as monitors, syringe pumps, ventilators, suction devices, defibrillator and gas cylinders. For safety reasons, this equipment should be mounted below the level of the patient. Battery life of all electronic devices should be at least several hours and battery life expectancy should be displayed. Of course all equipment must be lightweight and suitable for transfer conditions.

These types of transfer trolleys are usually bigger than standard ambulance stretchers, but during transfer the critically ill patient must be accessible from all sides. Therefore, these patients are commonly transferred in oversized, sometimes specially designed ambulances.

## Training

No studies have evaluated the effect of specific transfer training on outcome. However, since evidence shows that training for relatively simple procedures leads to quality improvement [70,71], it seems logical this applies also to more complex procedures such as transfer of critically ill patients. Transfer teams should be trained before taking responsibility for patient care during transport [22] and a significant determinant of quality of care during transport is the training of the attendant [37]. However, surveys continue to demonstrate a lack of formal training in transfer medicine [72,73].

Although local training initiatives have been described [74,75], intensive care societies have not implemented national training programmes. However, they all agree on the importance of specific transfer training for the transport team [14,15,56,58,76,77].

## Organizational and legal aspects

The decision to transfer a patient to another hospital must be made by the responsible consultant, in conjunction with consultant colleagues from relevant specialties in both the referring and receiving hospitals [15]. Ideally, the most appropriate receiving hospital is chosen and the patient or relatives agree with the transfer. Unfortunately, this is not always the case. Stakeholders do not always agree on the reasons for critical care transfers [78] and the most appropriate receiving hospital is not always chosen as the destination [79]. In the United Kingdom critical care networks have been established to improve this and to facilitate and organise transfers. Each network has a lead clinician and manager whose responsibilities include the development of referral pathways and transfer protocols [15]. Moreover, all acute hospitals must have systems and resources in place to resuscitate, stabilise and transport critically ill patients when required. They must have nominated a lead consultant for critical care transfers with responsibility for guidelines training and equipment provision [15]. The situation in the Netherlands is similar to that in the United Kingdom. All hospitals are expected to be able to transfer a high urgency critically ill patient using their own personnel. If time is not a critical factor, however, ICU patients should be transferred by a specialized retrieval team [80]. Interhospital transfer in Australia and New Zealand is arranged the same way as in the Netherlands. Transport of the critically ill patient has to be executed by a specialized retrieval team, including a medical practitioner. In the United States the situation differs. Due to insufficient regulatory control, these transfers are not so well organized [81]. Distribution of critical transfer teams, response time and transfer team composition differ around the country. In fact most often the teams consist of a nurse, paramedic and driver/pilot, although specially trained retrieval teams do exist [81]. In Canada, critical transfer teams often also operate without a physician. However, involved paramedics are well trained, so-called critical care paramedics, experienced in emergency, critical care and transport medicine.

In our opinion, a physician, preferably an intensivist, skilled and trained in the care of critically ill patients during transfer, should accompany the patient and be responsible for the care of the patient during the transfer. Therefore, although referring and receiving staff may agree on the transfer, the accompanying physician should be responsible for the final decision whether the patient is transferrable or not, and for treatment during the transport.

The moment at which responsibility transfers from one team to another should be clear to all involved, and should be stated in a regional or national transport protocol. A formal handover from referring doctor to retrieval doctor and from the latter to the receiving physician is therefore essential.

Since a transfer is a continuation of a patient’s treatment and since it is also a situation prone to incidents, it is of the utmost importance to document the transfer process. The clinical record should document the patient’s clinical status before, during and after transport, relevant medical conditions, environmental factors and therapy given. Moreover, organisations involved in medical transport should have an effective quality management system that can monitor and audit performance and make recommendations for appropriate improvements [15,56].

## Recommendations for the future

Further study of interhospital transfers is necessary but challenging for several reasons.

First, transfer-related incidents should be defined because the reported rates are partially a result of different definitions. Second, we have to agree on how transfers should be assessed. Long-term endpoints such as length of stay or mortality are probably difficult to evaluate and are of questionable relevance, since a transfer generally covers a small portion of the total ICU treatment. Changes in vital parameters just before and after transfer might be more reasonable, but then definitions or cut off points should be agreed. Stabilization prior to the transfer by the transfer team should also be taken into account. Third, case mix variation is difficult to control for since risk scores have not been developed for secondary transfers. Fourth, results may be influenced by different characteristics of specialized retrieval teams, such as team composition, whether or not a team received specific training and the kind of equipment used. It might be impossible to investigate the effect of all these characteristics separately. Fifth, although sicker patients are more likely to deteriorate during transfer [4], and thus may benefit more from a specialized transfer than less sick patients, we still have to define which groups of patients should be transferred by specialized retrieval teams. Sixth, in some countries it is already standard procedure to transfer the critically ill with a specialized transfer team, which may jeopardize further randomized controlled trials.

A randomized controlled trial, needed to provide the final evidence, is therefore probably only within reach when it is supported by our specialist societies and conducted in several countries simultaneously. In the interim, and on the basis of the available evidence, we feel that it is warranted to follow the recommendations/opinions listed in Table 2.

**Table 2:** **Recommendations for the transfer of the critically ill**

**Recommendations and opinions**	
•	Critically ill patients should be transferred by a specialized retrieval team
•	Intensive care should not be interrupted by transportation of the patient
•	Specialized retrieval teams should receive transfer training
•	Specific training programmes should be developed
•	Specialized retrieval teams should be staffed by a physician, preferably an intensivist and an ICU nurse
•	The accompanying physician makes the final decision whether the patient is transferrable and which treatment is given during the transport
•	Experience and training are more important than speed
•	Transfer organisations should have a quality management system
•	Incident reporting should be standardized and mandatory
•	Equipment used should conform with both ICU and transfer standards
•	Adults can learn from children (in the organization of transport)

## Conclusion

Over the past 40 years the quality of critically ill patient transfers has improved. Currently, it is increasingly accepted that these patients should be transferred by specialized retrieval teams, although definitive evidence is lacking. Further studies are necessary to provide this evidence. In short, we are on our way, but we are not there yet.
